# Access to Sexual Rights for all People with Disabilities: The Need to See and Include the Experiences of People with Intellectual Disability

**DOI:** 10.1007/s10508-023-02577-8

**Published:** 2023-04-13

**Authors:** Patsie Frawley

**Affiliations:** Te Kura Roi Tangata–Division of Education, University of Waikato, 100 Knighton Road, Hamilton, 3216 New Zealand

In their Target Article, Benoit et al. (2022) state that disability is “an umbrella term for impairments, activity limitations and participation restrictions” (World Health Organization, 2002). While definitions of disability can differ across countries, the World Health Organization (WHO) and the United Nations through the United Nations Convention on the Rights of Persons with Disabilities (UNCRPD) promote an understanding of disability that recognizes “Persons with disabilities are part of human diversity” (World Health Organization, 2022, p. 3). Importantly, the UNCRP also promotes that “disability” is an evolving concept (UN, 2006) meaning it is not fixed and can alter depending on the “prevailing environment from society to society” (United Nations, 2023).

In this Commentary, I refer to the understanding of disability as “an umbrella term” as outlined above and as applied by Benoit et al. (2022) as a dedifferentiated understanding of disability (Banks et al., 2020), which I argue does not apply well to the consideration of sexual rights for all people who identify or are labeled as “living with a disability,” in particular people with intellectual disabilities. Schaaf (2011) suggests that using such a definition of disability in relation to sexuality experiences is problematic and “obscures heterogeneity.” People with intellectual disability represent 20 percent of the world population of people with disabilities, which according to the World Health Organization (2022) is 1.3 billion people.^1^ Intellectual disability is broadly understood as the interaction between limitations of intellectual functioning which might include difficulties with learning, problem solving and judgment, and limitations of adaptive behavior which can include conceptual, social, communication, and practical skills (American Psychiatric Association, 2023).

The evolving understanding of intellectual disability has to a large extent stemmed from the intellectual disability self-advocacy movement that for more than four decades has called for a voice in the politics and policy that shapes their lives and has challenged the deficit-focused, diagnostic way intellectual disability is defined (Ellem et al., 2022). For this movement, self-identifying as a person with an intellectual disability and collectively voicing their experiences as people with intellectual disability differentiated from the generic experience of “Person Living with Disability” has been important because of the differentiated and notably restrictive policies and practices that have framed their experience of disability and of sexuality. These include institutionalization (Crossmaker, 1991; Johnson, 1998; Mirfin-Veitch & Conder, 2017), segregated special education (Akdemir, 2022; Balanoff & Wappett, 2013; Bouic, 2021; Frawley & Wilson, 2016; Nelson et al., 2020), restrictive practices including medications and chemical restrictions to address “challenging” sexual behaviors (Boer et al., 2011; Gates, 2019; Stein & Dillenburger, 2017), higher rates of sexual abuse (Bowen & Swift, 2017; Gil-Llario, et al., 2018), and sterilization (Rowlands & Jean-Jacques, 2017; Tilley et al., 2012). These experiences are inherently linked to how the sexuality of people with intellectual disability has been perceived, understood, and responded to in law, policy, and practice that have been informed by sex-negative (Williams et al., 2015) social scripts about intellectual disability and sexuality. While Benoit et al. (2022) have referred to a range of sex-negative scripts they found that applied to the sexuality of PLWD, their Target Article did not differentiate for the “particular” sex-negative scripts that frame the sexuality of people with intellectual disabilities. These scripts I suggest need to be seen in light of the differentiated experiences of sexuality of people with intellectual disability, and the barriers they face in attaining their sexual rights and sexual citizenship.

Accessing first person accounts in narrative and inclusive research is an important way of “hearing the experiences” of people with an intellectual disability (Black & Kammes, 2019; Frawley & O'Shea, 2020; Johnson et al., 2002; Luskie Murphy et al., 2019; Milner et al., 2019; O'Shea & Frawley, 2020b; Schaafsma et al., 2017), and is an emerging area of research that is often overlooked when research, including systematic and other literature reviews, fails to differentiate for disability experiences, as is evident in the review by Benoit et al. (2022). The remainder of this commentary will challenge if the umbrella term “People Living with Disabilities” (PLWD) is adequate for framing sexuality and disability research and understanding access to sexuality rights.

## To Differentiate or Not to Differentiate in Disability Sexuality Research?

Differentiation of experiences of disability and sexuality for people with an intellectual disability, as suggested by this Commentary, do need to be considered and highlighted in the research about accessing sexual rights, to ensure that these experiences are seen and understood, both in the ways they are similar to and how they differ from people with other lived experiences of disability. Research has found that promoting a dedifferentiated model of disability policy can disadvantage people with intellectual disabilities, particularly in terms of the overshadowing of what access means for people with an intellectual disability in comparison with people with other experiences of disability (Banks et al., 2020). In relation to the work of Benoit et al. (2022), the lack of differentiation in their approach to searching the literature about sexual assistance suggests a position that sees disability sexuality as a homogenous experience. This does not follow through, however, when looking at the way sexual rights are framed for, and experienced by, people with intellectual disabilities, tethering intellectual disability to inherent incompetence in sexual decision making, and seeing people with intellectual disability as inherently sexually vulnerable (Gill, 2015).

These “sex-negative” scripts are highlighted in the stories people with intellectual disabilities tell about their lived experience of sexuality in the research cited earlier. In particular, this research suggests for many people with intellectual disabilities, it is the mediation of their sexual lives and opportunities by others that limits their access to sexual rights. Research has found that the sexuality of people with intellectual disabilities is limited by the attitudes and practices of paid support staff and teachers (Deffew et al., 2022; Wilson & Frawley, 2015), families (Brown & McCann, 2019; Rushbrooke et al., 2014), and for some legal guardianship (Enujioke et al., 2021; Glen, 2019). Negative social attitudes about intellectual disability underpin these views (Dell'Armo & Tasse, 2021; Scior, 2011) and contributes to a hierarchy of disability (Deal, 2010) where people with intellectual disability are at the lower end of the hierarchy, particularly in relation to sexuality (Gill, 2015). Irish survey research in 2001, 2006, and 2011, for example, found that more people recognized the sexuality and sexual rights of people with physical or sensory disabilities than people with intellectual disability, including the right to have children (McConkey & Leavey, 2013).

Narrative research with people with intellectual disabilities, including Australian studies and a New Zealand study using similar methods enabled people with intellectual disabilities to tell stories of “loving and living” with partners of their choice, and where they enjoyed and highly valued their sexual relationships (Johnson et al., 2002; Luskie et al., 2019; O'Shea & Frawley, 2020a). These sex-positive scripts challenge the practices of those who limit the sexual opportunities of people with intellectual disability by claiming and showing sexual agency and reinforce the importance of hearing from people with intellectual disabilities about their successes in conducting sexual lives of their choice.

While many self-advocates look to the UNCRPD to remedy the social scripts limiting their sexuality rights, for people with intellectual disability, limitations on rights remain and are entrenched even in the UNCRPD.

## All Sexual Rights “For All”?

Benoit et al.’s (2022) use of Simon and Gagnon’s (1986) sexual script theory could also be applied to the way “discourses in society” have influenced and severely limited the sexual expression of people with intellectual disabilities. These “specific” discourses align strongly with the social fear and moral panic of reproducing people with intellectual disabilities that has underpinned oppressive, restrictive practices that equate to human rights violations in the lives of people with intellectual disability historically and currently. Canadian disability researcher and advocate Stainton (2017a) writes that what has underpinned the continuation of these practices over time has been the equating of intellectual disability with “non-humanness.” Stainton asks, “What lies at the heart of their [people with intellectual disabilities] otherness and why has it been such a consistent and potent force for oppression? Quite simply, intellectual disability strikes at the very heart of classical and modern ideas of value and humanness.” Stainton reflects on the centrality of “reason” to claims of humanness and citizenship informed by Western philosophers including Plato and Aristotle, and in a more modern context, Locke. The “ability to reason” using this philosophical stance is strongly valued and equated to humanness. When reason is contested as it is when “intellectual functioning” is determined to be limited, it follows that “humanness” is contested. Stainton (2017b) summarizes this as, “those that lack [reason]…will naturally lack nobility and value…and be [seen as] something less than human.” Stainton’s work has argued that it is this philosophical stance that is entrenched in Western laws and policy that has framed the oppressive, restrictive, and discriminatory practices that have shaped the lived experiences of people with intellectual disability.

Despite there being strong advocacy for sexuality rights to be clearly articulated as a right in the UNCRPD (Schaaf, 2011), opposition based on the generality of this right to all people with disabilities caused concerns. This was particularly so for countries that have laws and cultural and religious practices that have specific restrictions in place that apply to people with an intellectual disability, including sterilization laws and marriage customs and laws. Ruiz (2017) notes that “not all individuals of different gender identities, sexual orientations and disabilities have been conceived of as rights holders,” and “The silence on affirmative sexual and reproductive rights [in the UNCRPD] reinforced prejudices that equate disability with incompetence, incapacity, impotence and asexuality” (p. 94). These characteristics are notably aligned with definitions of intellectual disability.

Richards et al. (2009) noted that these were the same characteristics that were applied to support eugenics where in countries like the US involuntary sterilization of people with mental retardation [sic] was made law (see Buck v.Bell, 274 U.S 200 1927). Richards et al. argued that while these laws in some countries have been revoked, informed in some part by the UNCRPD, practices remain that are firmly based on views that people with intellectual disabilities are inherently “unable” to be sexual in the same way as “others.” This includes continuation of practices that control the reproductive rights of women with intellectual disabilities (McConnell & Phelan, 2022), restrictions on having consensual intimate sexual lives in residential services (Muswera & Kasiram, 2019), and practices that limit expression of sexual diversity by people with intellectual disabilities (Dinwoodie et al., 2016).

## Sexual Citizenship For “All”?

Gill (2002) frames the sexual lives of people with intellectual disability as “extraordinary” where it is deemed appropriate for their sexuality to be without intimacy, choice, and diversity. This Gill argued is based on the use of mental age in the psychological assessment of intellectual disability which leads to “childlike” connotations such as naivete and gullibility being attributed to the identity of people with intellectual disability framing them as incapable of “adult” sexual agency. Gill (2015) also asks in their book *Already Doing It: Intellectual Disability and Sexual Agency* whether sexual citizenship should depend on IQ levels.

Gill refers to these approaches and beliefs about sexuality and intellectual disability as a type of “sexual ableism” and discusses how this is framed specifically around the diminished expectations and opportunities that mediate the sexuality of people with intellectual disability. Importantly for this Commentary, this leads on to the reasons why we need to look at questions about equal sexuality rights for all people with disabilities, including people with intellectual disability where sexual intimacy relies on sexual assistance. This area of sexuality support is aligned with two articles of the UNCRPD; Article 25 the Right to access health services, which is inclusive of sexual health services and Article 23 Respect for Home and Family, which sets out the task of “eliminating discrimination against persons with disabilities in all matters relating to marriage, family, parenthood and relationships” (UN, 2006). Article 23 Respect for Home and Family further stipulates that State Parties must ensure “the means necessary to enable them to exercise these rights” (UN, 2006). However, this area of sexual support is highly contentious for people with intellectual disabilities despite progress for people with other lived experiences of disability as reviewed by Benoit et al. (2022) in their Target Article. Again, while ethical issues focus on questions of informed consent and decision making, there remains a possible “moral panic” about extending these services to people with intellectual disabilities.

While sexual assistance services for people with disabilities have been established in a number of countries, including Australia, Denmark, Britain, and Switzerland (Geymonat, 2019), the question of how inclusive they are and can be of people with intellectual disabilities has not been asked or reviewed in research. Geymonat highlights that the direction needed in this area is “alliance” of disabled people’s rights and advocacy with sex worker rights and advocacy. It is essential that the voices of people with intellectual disability and their grassroots self-advocacy is involved in these alliances. In Britain, the program “Supported Loving” ran through the disability support organization Choice Support (2023), which recognizes that people with intellectual disabilities may need and want various levels of support and assistance to develop and maintain relationships, including intimate relationships. This program offers a network of support for people with intellectual disabilities who do research and advocacy around sexuality rights, training for support staff and organizations, and has a range of co-developed resources on topics including aids and equipment, consent, contraception, abuse and safety, and information for support organizations on policy and practice. This holistic approach recognizes that sexual expression for people with intellectual disabilities requires a broad approach that might include physical support and assistance, but also includes educational needs and cultural change in organizations that support people with intellectual disabilities. This change needs to include a sexual rights perspective that differentiates what access to these rights means for people with intellectual disability whose sexual decision-making capacity is often contested.

## Conclusion

The Target Article by Benoit et al. (2022) notes that the majority of the articles they reviewed had a focus on the philosophical and moral issues associated with the attainment of sexual rights for PLWD. The Target Article encompassed a systematic literature review which used a broad and homogenous grouping of disability in its literature searching and reporting. In this Commentary, I have posed some questions about the appropriateness of this approach when the focus was on attainment of sexual rights and in particular what barriers there are to attaining the right to “be” sexual. Furthermore, in this Commentary, I have referred to research that highlights the “particular” experiences of people with intellectual disability as reported by them, and by research that has been framed by questions about the differentiated experience of intellectual disability and sexuality. This work, I argue, sheds a light on particular philosophical and moral questions about sexuality rights for people with intellectual disabilities that continue to impact laws, policy, and practice that limit the sexual citizenship of people with intellectual disabilities.

## Footnotes

1 The term Intellectual Disability is used in many Western countries. It replaced the term Mental Retardation in the US where often Intellectual and Developmental Disability is used. In the UK, intellectual disability is often used interchangeably with learning disabilities. The term Learning Disability is more commonly used in New Zealand. Generally in these countries the term refers to limitation in intellectual functioning and adaptive skills (see AIDD https://www.aaidd.org/intellectual-disability/definition; National Library of Medicine https://www.ncbi.nlm.nih.gov/books/NBK332877/).
